# Simulated Aging Studies on Porcelain Restoration Adhesives for Conservation in Chinese Museums

**DOI:** 10.3390/ma19040808

**Published:** 2026-02-20

**Authors:** Kaixun Chen, Guanqun Xu, Kai Wang, Maolin Zhang, Yanting Zhong, Feng Yuan, Zihan Li

**Affiliations:** 1 Institute of Archaeology, Cultural Heritage, and Chinese Civilization, Nanjing University, Suzhou 215163, China; 2 Ancient Ceramic Research Centre, Jingdezhen Ceramic University, Jingdezhen 333000, China; 3 Cultural Relics Protection Institute, Culture and Tourism Bureau of Yuhuatai District, Nanjing 210012, China; 4 Department of General Affairs, Nanjing University, Nanjing 210023, China

**Keywords:** porcelain restoration, conservation, simulated aging, Chinese museums

## Abstract

**Highlights:**

**What are the main findings?**

- Epoxy adhesives show high hardness but significant yellowing after simulated aging.
- Paraloid B-72 maintains excellent color stability but shows limited thermal stability.
- Cyanoacrylate 502 resists discoloration yet suffers embrittlement and interfacial failure.
- FTIR reveals distinct aging mechanisms among epoxy, acrylic, and cyanoacrylate adhesives.

**What are the implications of the main findings?**

- Adhesive selection must balance mechanical strength, color stability, and aging risks.
- Epoxy resins require caution due to aging-induced discoloration in visible repairs.
- B-72 remains suitable where reversibility and visual stability are priorities.
- Cyanoacrylate is better suited for temporary assembly than long-term conservation.

**Abstract:**

The rapid development of archaeology in China has led to the excavation of numerous fragmented porcelain artifacts, for which adhesive materials play a critical role in conservation and restoration. The long-term stability of these adhesives directly affects the structural safety and visual integrity of restored objects. In this study, four adhesives widely used in Chinese conservation practice—epoxy resin Hezhong AAA, epoxy resin Hongxing 509, acrylic resin Paraloid B-72, and cyanoacrylate adhesive 502—were systematically investigated through simulated cyclic aging experiments. A multi-analytical approach was employed, including ultra-depth-of-field microscopy, CIE Lab* colorimetric analysis, pencil hardness testing, and Fourier transform infrared spectroscopy (FTIR). The results reveal distinct aging behaviors among different adhesive types. Epoxy resin adhesives exhibit high initial hardness and pronounced hardening during aging, with coating hardness increasing from the B range to the H range after 15 aging cycles; however, they also show significant yellowing, with total color differences (ΔE) exceeding 10 and dominated by increases in the b* parameter. Paraloid B-72 maintains excellent color stability throughout aging, with ΔE values consistently below 2, although it shows limited thermal stability and delayed physical stabilization. The cyanoacrylate adhesive 502 demonstrates rapid curing and minimal discoloration but undergoes embrittlement and interfacial debonding during aging, indicating reduced long-term bonding reliability. By correlating macroscopic performance evolution with molecular-level chemical changes, this study elucidates the aging mechanisms of commonly used restoration adhesives and provides a scientific basis for adhesive selection, risk assessment, and long–term preservation strategies in porcelain conservation.

## 1. Introduction

Porcelain archaeological artifacts constitute a significant proportion of unearthed cultural relics in China. With the continuous advancement of archaeology in recent years, a large number of excavated porcelain artifacts have successively entered the stages of conservation and restoration. In the practice of porcelain artifact restoration, adhesives have become indispensable as key materials due to their extensive application in processes such as fragment reassembly, structural stabilization, and morphological reconstruction [1,2,3]. Once adhesives are applied to cultural relics and enter the long-term preservation phase, their performance inevitably deteriorates over time. Porcelain, as an inorganic material primarily composed of silicates, exhibits significantly superior aging resistance compared to organic polymer materials [4]. Over an extended temporal scale, adhesives tend to undergo deterioration in physical properties and chemical structural changes before the porcelain substrate itself, thereby becoming the most vulnerable component within the restoration system.

To evaluate the reliability and stability of materials over extended periods of use, artificial accelerated aging tests are commonly employed as a critical reference method [5,6,7,8]. In the field of international cultural heritage conservation, a relatively systematic research framework for aging experiments has been established for commonly used conservation and restoration materials, accompanied by a substantial accumulation of experimental data. For instance, epoxy-based adhesives such as Hxtal NYL-1 [9,10,11,12,13] and acrylic resins like Paraloid B-72 [14,15,16,17,18] have undergone comparative studies involving long-term artificial aging and natural aging under varying conditions of light, temperature, humidity, and chemical environments. Their performance in terms of mechanical property changes, yellowing behavior, molecular structural stability, and reversibility has been thoroughly characterized through extensive experimentation. These investigations furnish scientific evidence supporting the suitability of such materials for use in cultural heritage conservation and form an essential foundation for internationally recognized standards in material selection and evaluation.

In contrast, conservation practice in China relies heavily on domestically produced adhesives that are readily available and cost–effective, such as Hezhong AAA epoxy resin [19,20,21,22,23,24,25,26,27,28], Hongxing 509 epoxy adhesive [26,29,30,31], and cyanoacrylate-based Beijing Chemical Works 502 adhesive [22,25,29,32,33,34,35,36]. Although these materials are widely used in museums and archaeological fieldwork, their selection is often based on empirical experience rather than systematic material evaluation. Comprehensive and comparable studies on their aging behavior, physicochemical stability, and long-term risks remain scarce, particularly in the international literature. This lack of scientific assessment limits objective decision-making and may compromise the long-term safety of restored porcelain artifacts.

From a materials perspective, epoxy and acrylic adhesives differ fundamentally in their curing mechanisms and performance profiles. Epoxy resins are thermosetting polymers that form highly cross-linked three-dimensional networks through reactions between epoxy groups and curing agents [37], resulting in high mechanical strength and good chemical resistance [38,39]. However, their dense cross-linked structures and aromatic backbones often render them vulnerable to photo-oxidative discoloration and increased brittleness during aging [40,41,42]. Acrylic resins, by contrast, are typically thermoplastic polymers that cure through solvent evaporation or polymerization of acrylate monomers [14]. They generally exhibit better optical stability and reversibility but comparatively lower mechanical strength and thermal resistance [17,43]. Cyanoacrylate adhesives cure rapidly through anionic polymerization and are operationally convenient [44], yet their long-term stability and interfacial durability under museum environments remain questionable [45].

To elucidate the chemical degradation mechanisms of adhesives during aging, vibrational spectroscopic techniques—particularly Fourier transform infrared spectroscopy (FTIR) and Raman spectroscopy—have been widely employed for chemical structure identification and aging-process monitoring [46,47,48,49]. These methods are advantageous due to their non-destructive or minimally invasive nature and high chemical sensitivity, enabling the evolution of functional groups and molecular rearrangements to be tracked through changes in band intensity, position, and shape [11,50]. Such spectral variations provide critical evidence for key aging reactions, including oxidation, hydrolysis, photodegradation, chain scission, and changes in crosslinking density. For example, in FTIR spectra, the enhancement of carbonyl (C=O) absorption, the emergence or broadening of hydroxyl (O–H) bands, and variations in C–O–C vibrational features are commonly used to indicate the formation of oxidation products and ester hydrolysis pathways [51,52,53]. In contrast, Raman spectroscopy offers complementary advantages in identifying aromatic structures, conjugated double bonds, and certain additives, thereby assisting in the interpretation of molecular backbone evolution and conjugation changes during aging [49,54]. Overall, the integrated analysis of multi-spectroscopic evidence not only facilitates the identification of dominant failure modes and vulnerable degradation pathways in aged adhesives but also provides a more targeted scientific basis for material suitability assessment, risk evaluation, and formulation optimization.

Against this background, the present study systematically investigates the aging behavior of four representative adhesives widely used in Chinese porcelain conservation practice: two epoxy resins (Hezhong AAA and Hongxing 509), two acrylic resins (Paraloid B-72 and Beijing Chemical Works 502). Through a multi-analytical approach combining ultra-depth-of-field microscopy, colorimetric analysis, pencil hardness testing, and Fourier transform infrared spectroscopy (FTIR), this work aims to (i) characterize the aging-induced physical and chemical evolution of these adhesives, (ii) compare their relative advantages and limitations from a conservation-oriented perspective, and (iii) propose a functionally differentiated adhesive selection strategy that balances structural stability, visual compatibility, and long-term conservation safety. By focusing on materials commonly used in Chinese museums yet insufficiently documented in international research, this study seeks to bridge the gap between empirical practice and evidence-based conservation science.

## 2. Materials and Methods

### 2.1. Materials

Based on the foundational survey and literature review regarding adhesives commonly used in the restoration of Chinese porcelain artifacts, this study further selected four commercially available adhesives as representative research subjects. The types and fundamental characteristics of these adhesives demonstrate typicality and a foundation in practical application. Detailed information on the selected adhesives is presented in Table 1.

**Figure 1:**
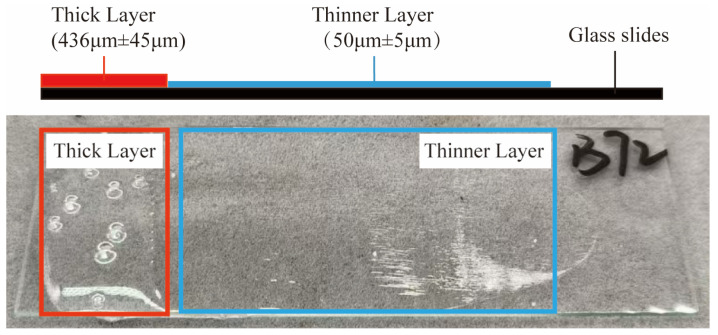
Schematic illustration of sample preparation on glass slides, showing two functional coating regions with different thicknesses: a thinner layer (50 μm ± 5 μm) used for colorimetric analysis and pencil scratch hardness testing, and a thicker layer (436 μm ± 45 μm) used for microscopic observation and Fourier transform infrared (FTIR) analysis.

The four adhesives were uniformly applied onto microscope slides (76.2 × 25.4 mm) using an SZQ coating applicator (Shanghai Qigong Instrument Equipment Co., Ltd., Shanghai, China), with the coating thickness controlled at 50 μm. The specific procedure involved dispensing an appropriate amount of adhesive onto one end of the slide and then steadily moving the applicator to spread the adhesive into a uniform layer, with excess adhesive accumulating at the opposite end. The coated samples were left to stand in a fume hood for 72 h to ensure complete curing before testing and cyclic aging experiments. This coating procedure resulted in the formation of two functional regions with different thicknesses: a thinner layer, with an average thickness of approximately 50 μm (standard deviation, SD ≈ 5 μm), designated for colorimetric analysis and pencil scratch hardness testing; and a thicker layer, formed by the accumulation of excess adhesive during the coating process, exhibiting an average thickness of approximately 436 μm (SD ≈ 45 μm), which was used for microscopic observation and Fourier transform infrared (FTIR) analysis. The coating thickness was determined by optical microscopic observation, with measurements taken at multiple positions along each coating region. The average thickness and SD were calculated to characterize the thickness uniformity of the adhesive layers (Figure 1).

### 2.2. Design of Cyclic Aging Experiments

To simulate complex aging environments, this study employed cyclic aging tests to systematically evaluate the performance of the adhesives. The cyclic aging protocol consisted of three stages:

(1) UV aging simulation: Samples were placed 4 cm from the light source of a ZF-23Max UV analyzer (Shanghai Xinulibo Instrument Co., Ltd., Shanghai, China) and exposed to UV radiation at wavelengths of 254 nm and 365 nm (average UV intensity: 44.0 mW/cm^2^) for 12 h.

(2) High-temperature, low-humidity aging simulation: Samples were placed in a ZH-AXD-150 aging chamber (Dongguan Zhenghang Instrument Equipment Co., Ltd., Dongguan, China) under conditions of 80 °C and 20% relative humidity for 12 h.

(3) High-temperature, high-humidity aging simulation: Using the same aging chamber, samples were subjected to 50 °C and 90% relative humidity for 12 h.

Each full aging cycle lasted 36 h, with a total of 15 cycles conducted, resulting in a cumulative experimental duration of 540 h. After each cycle, the microscopic surface morphology and color difference (ΔE) of the samples were recorded.

### 2.3. Characterization and Testing

#### 2.3.1. Surface Morphology Analysis Using Ultra-Depth-of-Field Microscopy

In this experiment, the surface morphology of the adhesives was recorded and analyzed after each cyclic aging interval using a VHX-6000 ultra-depth-of-field microscope manufactured by KEYENCE Corporation (Osaka, Japan). The observation magnification was set to 100×, enabling high-resolution imaging to capture the evolution of surface morphology during the aging process. By comparing images obtained after multiple aging cycles, the cumulative effect of aging on the micro-morphology of the adhesives could be visually assessed, providing scientific evidence for elucidating their aging mechanisms.

#### 2.3.2. Colorimetric Testing

To evaluate the impact of adhesive aging on visual appearance, this study employed an NF-333 colorimeter manufactured by Nippon Denshoku Industries Co., Ltd. (Tokyo, Japan) to measure the chromaticity values of the samples. The analysis was conducted in accordance with the CIE-Lab* color space standard established by the International Commission on Illumination (CIE) [55]. By measuring the chromaticity values of the samples before and after aging, the total color difference (ΔE) was used to quantify the degree of color change during the aging process. The ΔE value is calculated using the following formula:

(1) $$\Delta E=\sqrt{{\left(\Delta L^{*}\right)}^{2}+{\left(\Delta a^{*}\right)}^{2}+{\left(\Delta b^{*}\right)}^{2}}$$

where ΔL*, Δa*, and Δb* represent the changes in lightness (L*), red-green value (a*), and yellow-blue value (b*), respectively. In this formula, the L* axis describes the black-white distribution (0 indicates pure black, 100 indicates pure white); positive values on the a* axis indicate red tones, while negative values indicate green tones; positive values on the b* axis indicate yellow tones, while negative values indicate blue tones. A larger ΔE value indicates a more pronounced color difference. Additionally, after each cyclic aging interval, the chromaticity of the adhesive samples was observed and recorded. The changes in their L*, a*, and b* values were measured, and the ΔE between consecutive cycles was calculated to analyze the dynamic trend of color change during the aging process. By progressively documenting chromaticity variations across aging stages, the cumulative effect can be visually quantified, providing a scientific basis for evaluating the deterioration in visual appearance during adhesive aging.

Colorimetric measurements (L*, a*, b*, and ΔE) were performed on the same specimen at successive aging cycles in order to track the time-dependent evolution of color properties under cyclic aging conditions. As the ΔE values represent progressive changes in an individual sample rather than statistical distributions obtained from multiple independent specimens at a given aging stage, standard deviations were not calculated for ΔE at each cycle. This approach allows the aging trajectory to be clearly visualized without introducing statistically inappropriate dispersion parameters.

#### 2.3.3. Pencil Scratch Hardness Test

To comprehensively characterize the changes in the physical properties of adhesives during aging, this study employed pencil scratch hardness testing to quantify hardness [56]. The experiment utilized a QHQ-A pencil scratch hardness tester, with parameters set as follows: load accuracy of 750 g ± 10 g, scratch speed of 1.0 ± 0.1 mm/s, and a constant scratch angle of 45° ± 1°. An extended pencil hardness grading range (6B to 9H, totaling 19 grades) was adopted for testing, preferentially using Zhonghua brand 101 drawing pencils to ensure accuracy and consistency.

Hardness testing of the samples was conducted across five sampling stages: initial state (unaged), after the 4th aging cycle, after the 8th aging cycle, after the 12th aging cycle, and after the 15th aging cycle. At each stage, hardness values were recorded for the initial sample, first sample, second sample, third sample, and fourth sample, respectively. The results of the pencil scratch hardness tests provide a clear analysis of the evolution of adhesive hardness during cyclic aging, offering a scientific basis for evaluating the deterioration of their physical properties throughout the aging process.

Pencil hardness testing was employed here as a comparative indicator under identical testing conditions rather than an absolute mechanical property, allowing relative assessment of aging-induced stiffening among different adhesive systems.

#### 2.3.4. Fourier Transform Infrared Spectroscopy(FTIR) Analysis

This study employed Fourier transform infrared spectroscopy (FTIR) to characterize the chemical structure of adhesive samples and investigate changes in their functional groups during aging. A Nicolet 5700 spectrometer (Thermo Fisher Scientific, Waltham, MA, USA) was used for full-spectrum scanning within the wavenumber range of 400–4000 cm^−1^. To ensure sample representativeness, the adhesives were first cryogenically embrittled using liquid nitrogen and then ground into 200-mesh powder. Samples were prepared via the potassium bromide (KBr) pellet method (sample-to-KBr mass ratio of 1:100), with each spectrum acquired from 32 cumulative scans at a resolution of 2 cm^−1^.

During spectral acquisition and processing, background contributions from ambient water vapor and carbon dioxide were treated using the instrument’s built-in atmospheric compensation functions, with identical parameters applied to all spectra. As such, model-based corrections may become overly aggressive in the high-wavenumber region, particularly around ~3700 cm^−1^ associated with O–H stretching vibrations; the interpretation of this region was approached with caution. Accordingly, the discussion in this study emphasizes relative spectral evolution between pre-aging and post-aging samples, rather than absolute variations in individual peak intensities.

The analysis focused on changes in the chemical groups of the adhesives before and after aging. By comparing the FTIR absorption spectra of powdered adhesive samples pre- and post-aging, the evolution of functional groups was examined, thereby providing a scientific basis for a deeper understanding of the aging mechanisms of the adhesives. Considering the irregular sampling typical of adhesive materials in practical restoration work (often involving non-uniform fragments ultimately ground into powder), the adhesives were prepared in powdered form to enhance the reliability and representativeness of the experimental results.

## 3. Results

### 3.1. Morphological Observation via Ultra-Depth-of-Field Microscopy

**Figure 2:**
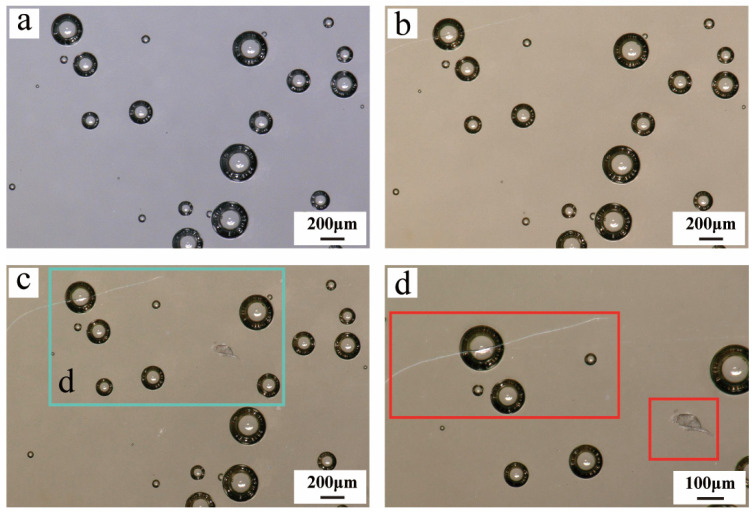
After undergoing aging cycles, the surface of Hezhong AAA adhesive gradually changed from its initial colorless and transparent state (**a**) to yellow and transparent with visible cracks (**b**), ultimately developing through-thickness cracks at bubble sites (**c**,**d**). Panel (**d**) is an enlarged view of the cyan boxed region in panel (**c**), and the red box in panel (**d**) highlights the cracks at bubble sites.

After undergoing 15 simulated aging cycles, the Hezhong AAA adhesive exhibited not only significant surface discoloration (Figure 2a,b) but also the formation of cracks on the surfaces of internal bubble regions within the adhesive (Figure 2c,d). Specifically, initial cracks began to appear at bubble sites on the adhesive surface after the 12th aging cycle (Figure 2b); by the 15th cycle, these cracks had further propagated and gradually extended across the entire bubble surface (Figure 2c,d). As the aging process advanced, the adhesive surface progressively hardened. The bubble regions, serving as structural weak points within the adhesive layer, became more susceptible to stress concentration under repeated aging cycles. This localized stress likely induced the initiation of micro-cracks, which subsequently continued to propagate during further aging cycles [57].

**Figure 3:**
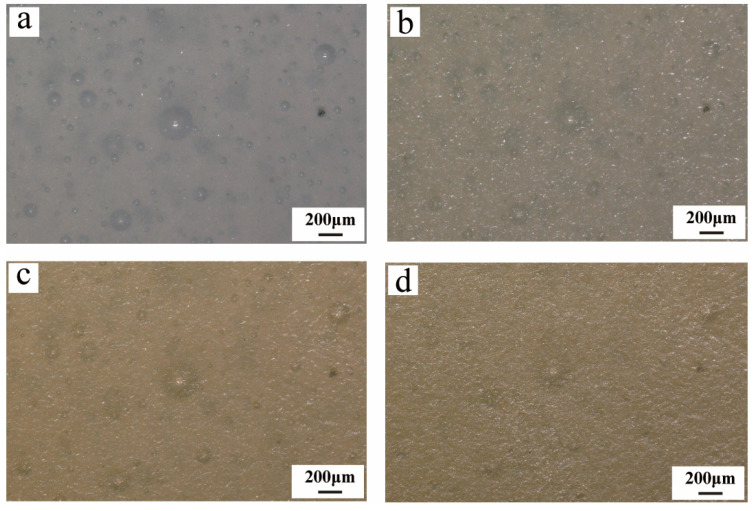
After undergoing aging cycles, the Hongxing 509 adhesive evolved from its initial milky white translucent texture (**a**) through progressive reductions in transparency and gloss (**b**) and surface roughening (**c**), ultimately forming an irregular, “orange peel”-like wrinkled structure (**d**).

The initial texture characteristics of the Hongxing 509 adhesive differ noticeably from those of Hezhong AAA. While Hezhong AAA presents a clean, transparent, and homogeneous gel-like morphology in its unaged state (Figure 2a), Hongxing 509 initially exhibits a milky white, translucent texture (Figure 3a). As aging progresses, the transparency and surface gloss of Hongxing 509 decrease significantly (Figure 3b). Its surface morphology transitions from relatively smooth to a rough state (Figure 3c) and ultimately develops an irregular, wrinkled structure resembling an “orange peel” texture (Figure 3d).

The observed phenomenon is closely related to the inherently poor leveling properties of this adhesive. In the pre-aged state, the orange-peel texture on the surface is not fully apparent due to the material’s remaining transparency. Instead, light scattering at the adhesive surface creates a diffused reflection effect, resulting in an overall whitish, translucent appearance. As aging continues, the adhesive’s transparency gradually diminishes, reducing its ability to transmit light. This enhances the surface scattering effect, making the initially concealed orange-peel texture progressively more visible and clearly distinguishable.

**Figure 4:**
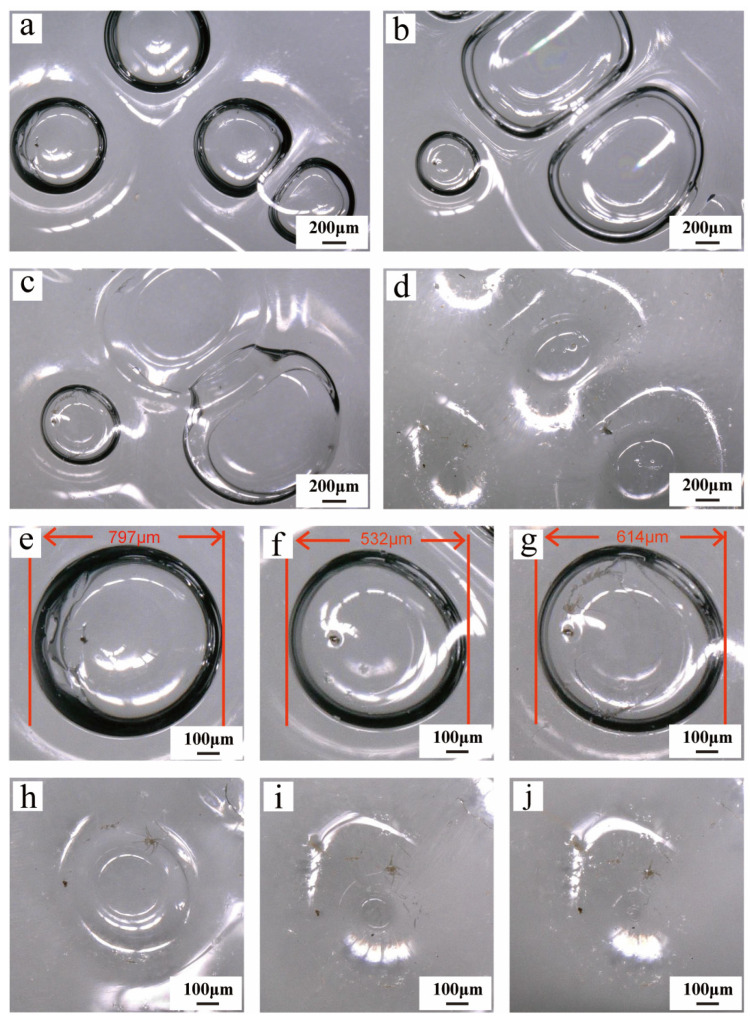
Morphological evolution of the Paraloid B-72 surface during aging cycles. In the initial stage, significant changes in bubble morphology occurred due to solvent evaporation (**a**,**b**), followed by stabilization and subsequent stages of cracking and softening (**c**), culminating in complete integration into the adhesive matrix (**d**). Detailed observation of a specific bubble shows its diameter decreasing from an initial 797 μm (**e**) to 532 μm after the first aging cycle (**f**), expanding to 614 μm upon cracking by the eighth cycle (**g**), rupturing by the tenth cycle (**h**), beginning to dissolve into the adhesive matrix after the twelfth cycle (**i**), and being fully integrated into the adhesive matrix after the fifteenth cycle (**j**).

Paraloid B-72, a representative acrylic adhesive commonly used in artifact conservation practice, was applied in this experiment as a 10 wt% solution in acetone. Significant changes in the morphology of surface bubbles were observed even in the early stages of the experiment (Figure 4a,b). This is primarily because Paraloid B-72 is supplied as solid pellets that require dissolution in an organic solvent prior to use. Even after initial drying, a certain amount of solvent that has not fully evaporated may remain within the adhesive layer. During the aging process, this residual solvent gradually evaporates, causing the adhesive layer to remain in a relatively fluid state for a period, which in turn leads to noticeable changes in the morphology of surface bubbles. As aging continues, from the second cycle onward, the solvent is largely evaporated, and the bubble morphology gradually stabilizes (Figure 4b). In subsequent aging stages, the surface bubbles undergo further processes of cracking, softening, and eventual integration into the adhesive matrix (Figure 4c,d). Detailed sequential observation of a specific bubble revealed the following evolution: its diameter decreased from an initial 797 μm (Figure 4e) to 532 μm after the first aging cycle (Figure 4f); it cracked and expanded to 614 μm by the eighth cycle (Figure 4g); it completely ruptured by the tenth cycle (Figure 4h); and by the fifteenth cycle, the bubble structure was fully integrated into the adhesive matrix (Figure 4i).

Although Paraloid B-72 exhibited no significant color change throughout the aging process, it still demonstrated a certain degree of instability under high-temperature conditions [58]. This observation indicates that the aging process has noticeably affected the physical stability of B-72, particularly in high-temperature environments where physical structural changes are likely to first occur in bubble regions formed during the bonding process and at irregular adhesive interfaces (Figure 4g,h). Such changes may further compromise the adhesive’s bonding performance and long-term stability, suggesting that in practical restoration and preservation, close attention should be paid to the thermal resistance and aging risks of Paraloid B-72 under elevated temperatures.

**Figure 5:**
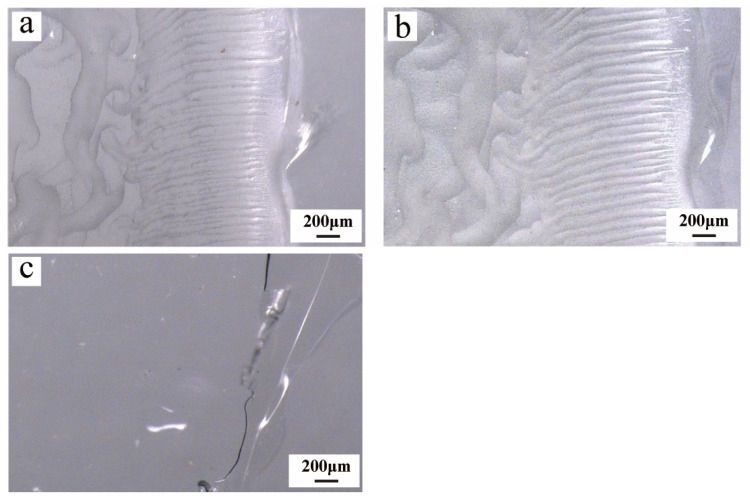
After aging cycles, the surface morphology of adhesive 502 showed no significant changes compared to its initial state (**a**,**b**). However, detachment and fracture phenomena between the thin adhesive layer and the glass slide were observed in the film region (**c**).

Beijing Chemical Works502 adhesive demonstrated relatively good stability in terms of post-aging morphological characteristics. After undergoing 15 cycles of aging experiments, no significant color change or pronounced morphological degradation was observed on the surface of its adhesive accumulation zone (Figure 5a,b). Judging from its macroscopic appearance, this adhesive maintained its initial state well throughout the aging process, exhibiting the most notable apparent stability among the four adhesives investigated in this study.

Despite the lack of significant changes in its surface morphology, a marked decline in adhesion performance between the adhesive and the glass slide was observed after completing the 15 aging cycles. Specifically, the adhesive in the thin-film region began to undergo embrittlement and fracture, with localized areas showing signs of lifting and delamination from the glass slide surface (Figure 5c). This result indicates that during aging, the degradation of interfacial bonding performance likely precedes any noticeable deterioration in macroscopic surface morphology. It suggests that in cultural heritage restoration applications, attention must be paid not only to the adhesive’s appearance but also to the stability of its bonding interface and the long-term risks associated with its use.

### 3.2. Colorimetric Testing

**Figure 6:**
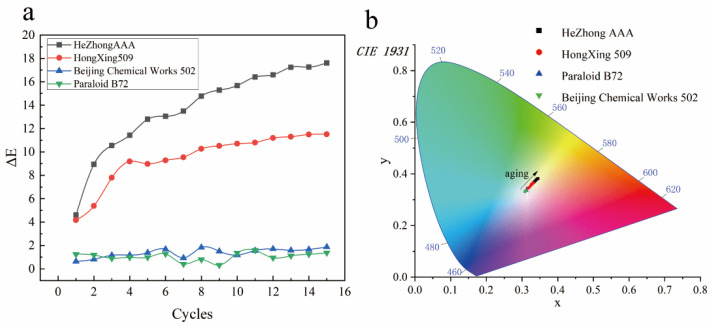
During the aging process, epoxy resin adhesives (Hezhong AAA and Hongxing 509) exhibited relatively pronounced changes in color difference, while acrylic resin adhesives (Paraloid B-72 and 502) showed minimal color variation (**a**). CIE 1931 chromaticity analysis indicates that the color changes in the adhesives were predominantly concentrated within the 570–580 nm yellow wavelength range, reflecting a commonly observed yellowing phenomenon (**b**).

The color variation results of the four adhesives after undergoing 15 cycles of aging experiments, totaling 540 h, are presented in Appendix A. These results illustrate the changes in the colorimetric parameter (ΔE) for each adhesive, reflecting the degree of visual degradation or alteration during the aging process. These chromaticity variations provide important reference data for assessing material stability and analyzing the influence of simulated environmental conditions on adhesive performance. After 15 aging cycles, significant differences were observed in the color variation behavior among the four adhesives (Figure 6a).

As epoxy resin adhesives, both Hezhong AAA and Hongxing 509 exhibited noticeable color changes at the onset of the first aging cycle (Figure 2 and Figure 3), with the discoloration intensifying progressively in subsequent cycles. Specifically, neither adhesive showed significant changes in L* values, whereas the b* values changed markedly. The b* value of Hezhong AAA increased from an initial 0.18 to 17.52, and that of Hongxing 509 rose from 0.58 to 11.43. This pronounced increase in b* values indicates a general shift toward a yellowish tone in both adhesives. Additionally, their a* values also changed to some extent, though the magnitude was relatively smaller. The a* value of Hezhong AAA decreased from an initial −2.57 to −5.56, while that of Hongxing 509 dropped from −2.45 to −5.22. During the first aging cycle, the a* values of the two adhesives already reached −4.22 and −3.82, respectively, and subsequently remained within the range of −4 to −6, with some fluctuations. Overall, the color shift trend primarily developed along the yellow visible light band (570–580 nm), reflecting the characteristic yellowing of the adhesives during aging (Figure 6b).

In contrast, the acrylic resin adhesive Paraloid B-72 and the cyanoacrylate adhesive Beijing Chemical Works 502 exhibited a high resistance to color change throughout the aging process. Visual inspection revealed no obvious discoloration (Figure 5 and Figure 6). Quantitative colorimetric data showed that variations in L*, a*, and b* values for both adhesives remained within ±2 relative to their initial values, with no systematic trend observed over successive aging cycles. Considering the inherent uncertainty associated with instrumental color measurements, these variations can be regarded as statistically negligible. Moreover, the ΔE values for both adhesives consistently remained below 2 during the entire aging period, confirming their excellent color stability under the simulated aging conditions (Figure 6a).

### 3.3. Pencil Scratch Hardness

After 15 cycles of aging experiments, all adhesive coatings exhibited varying degrees of increased hardness. This trend aligns with common observations of molecular structural changes in polymeric materials during aging. Specifically, polymer materials are subject to the combined effects of environmental factors such as light, heat, and oxygen during aging, which reduce the molecular mobility of polymer chain segments, decrease material elasticity, and concurrently increase cross-linking density, collectively manifesting as a gradual increase in hardness.

**Figure 7:**
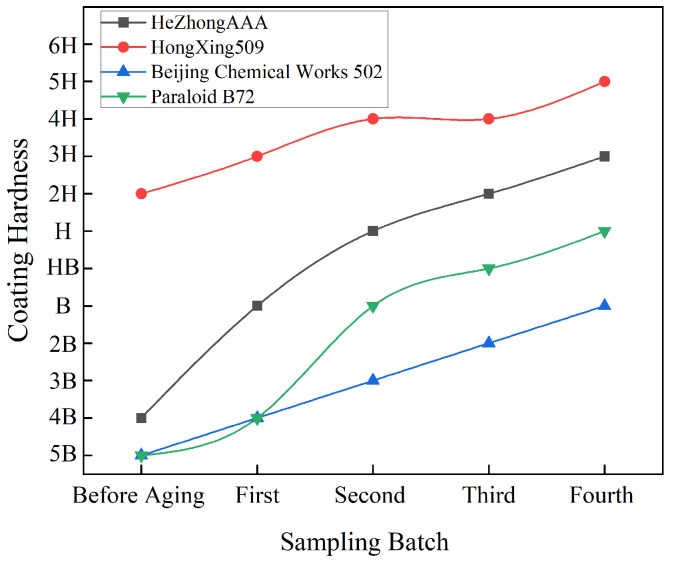
After 15 aging cycles, the hardness of all adhesive coatings increased, reflecting a general trend of enhanced molecular cross-linking density. However, significant differences were observed among the adhesives in their response: epoxy resins showed the most pronounced hardening, with Hongxing 509 exhibiting the highest hardness after aging. In contrast, the overall hardening degree of acrylic resins was lower than that of epoxy resins, with 502 displaying the smallest increase in hardness and ultimately the lowest final hardness.

Significant differences were observed in the responses of different adhesive types to the aging environment (Figure 7). The epoxy resin adhesive Hezhong AAA showed a clear hardening trend during cyclic aging. Its coating hardness progressively increased from a pre-aging level of 4B to approximately 3H at the fourth sampling interval, indicating a strong aging-induced hardening effect. Hongxing 509 maintained a high level of coating hardness throughout the aging process, with its hardness rising from an initial 2H to the 5H range in later stages, making it the adhesive with both the highest initial hardness and the highest post-aging hardness among the four.

The coating hardness of the acrylic resin Paraloid B-72 gradually increased from an initial 5B to H. Although a certain degree of hardness increase was observed, the overall magnitude of change was relatively limited. This relatively moderate hardening behavior is consistent with its molecular characteristics, which are dominated by linear or weakly cross-linked structures, allowing it to retain a degree of flexibility during aging. This makes it more suitable for restoration contexts requiring reversibility and deformation tolerance. The coating hardness of Beijing Chemical Works502 gradually increased from 5B to approximately B, showing a relatively smooth, nearly linear growth trend. However, its hardness increase was the smallest among the four adhesives, indicating a weaker response to the cyclic aging environment.

### 3.4. Fourier Transform Infrared Spectroscopy

After completing 15 cycles of aging experiments, Fourier transform infrared spectroscopy (FTIR) analysis was performed on the prepared samples of the four adhesives. The results indicate varying degrees of chemical structural changes and functional group evolution characteristics in the adhesives during aging.

**Figure 8:**
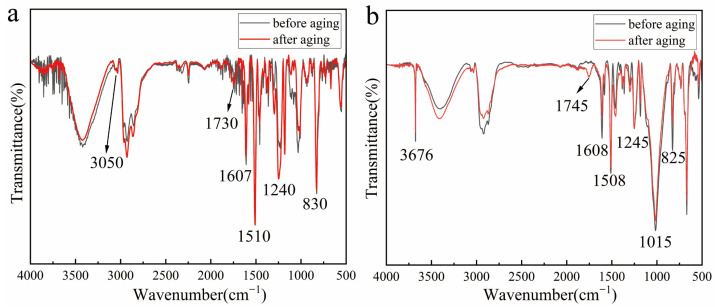
FTIR analysis revealed that both Hezhong AAA (**a**) and Hongxing 509 (**b**) generated carbonyl groups after aging, accompanied by noticeable yellowing. Among them, Hongxing 509, modified with silicone compounds, demonstrated superior chemical stability.

The comparative FTIR spectra of the Hezhong AAA adhesive before and after aging are presented in Figure 8a, with the corresponding functional group assignments and spectral changes summarized in Table 2. Prior to aging, absorption peaks at 1607 cm^−1^ and 1510 cm^−1^ correspond to the stretching vibrations of aromatic C=C bonds, confirming the presence of a bisphenol A backbone [59]. The out-of-plane bending vibration of para-substituted aromatic C–H bonds at 830 cm^−1^ further supports the existence of the bisphenol A aromatic structure. Therefore, structurally, Hezhong AAA is a typical bisphenol A-type epoxy resin. Absorption peaks at 1240 cm^−1^ and 917 cm^−1^ correspond to the asymmetric and symmetric stretching vibrations of the epoxy group (C–O–C), respectively, reflecting the integrity of the cross-linked network [60].

After aging, significant new peaks appeared in the FTIR spectrum. The new peak at 1730 cm^−1^ is attributed to the stretching vibration of carbonyl groups (C=O). In addition, an absorption band near 1650 cm^−1^ emerged after aging, which may be associated with conjugated carbonyl structures generated through oxidative modification of the bisphenol A aromatic rings. These oxidation-related chemical changes are closely linked to the gradual yellowing of the adhesive and the resulting alteration in surface color [53]. After aging, the intensities of absorption peaks at 1607 cm^−1^, 1510 cm^−1^, 1240 cm^−1^, and 830 cm^−1^ increased to varying degrees, indicating that the cross-linking process of the adhesive continued to deepen during aging. Notably, the positions of the major absorption peaks showed no significant attenuation, suggesting that despite undergoing aging, the cross-linked network of the adhesive retained strong structural stability.

In contrast, another epoxy resin adhesive, Hongxing 509, exhibited certain differences compared to Hezhong AAA. The FTIR spectra of Hongxing 509 epoxy adhesive before and after aging are shown in Figure 8b, with the corresponding functional group assignments and aging-induced spectral changes summarized in Table 3. In the unaged state, the characteristic peak at 1508 cm^−1^ is attributed to the C=C stretching vibration of the aromatic ring in the bisphenol A backbone, while the peak at 825 cm^−1^ indicates the out–of–plane bending vibration of para-substituted C–H bonds in the aromatic ring, further confirming the para-substitution characteristic of the bisphenol A structure. The asymmetric stretching vibration of the epoxy group (C–O–C) at 1245 cm^−1^ represents a core characteristic peak of the epoxy resin cross-linked network. Simultaneously, the stretching vibration of the siloxane bond (Si–O–Si) at 1015 cm^−1^ clearly indicates that the material has undergone silicone compound modification. Thus, it can be determined that Hongxing 509, like Hezhong AAA, is a bisphenol A-type epoxy resin, a silicone-modified bisphenol A epoxy adhesive.

After aging, the overall spectral framework of the epoxy network remained largely unchanged, as reflected by the comparable intensity of the C–O–C band at 1245 cm^−1^ (medium before and after aging). However, several aging-related chemical changes were evident. Most notably, a carbonyl band at ~1745 cm^−1^ increased from weak to medium intensity, indicating the formation of oxidation-induced carbonyl-containing species. Such oxidation products are commonly associated with the yellowing of epoxy materials during aging. Meanwhile, the out-of-plane aromatic C–H band at 825 cm^−1^ weakened (medium to weak), suggesting partial oxidation-related alteration of the aromatic structure and/or its substitution environment.

The stretching vibration peak of the siloxane bond (Si–O–Si) at 1015 cm^−1^ showed no marked changes before and after aging, indicating that the silicone-modified part did not undergo significant hydrolysis or oxidation [63]. The stability of the siloxane bonds significantly enhanced the material’s aging resistance under hygrothermal and oxidative conditions, helping to delay the degradation of the cross-linked network while maintaining, to some extent, the material’s mechanical properties and functionality.

**Figure 9:**
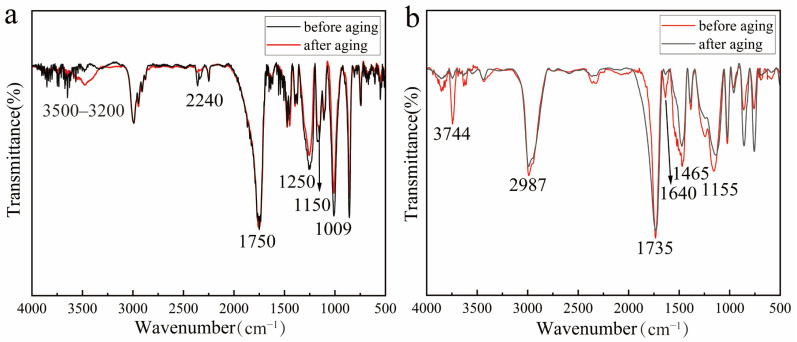
FTIR analysis reveals that Paraloid B-72 primarily undergoes ester group decomposition and polymer backbone cleavage after aging (**a**). In contrast, adhesive 502 experiences ester hydrolysis during aging, accompanied by intensified carbonyl and cyano peaks, leading to the formation of a dense cross–linked network. This results in increased hardness but reduced toughness (**b**).

The FTIR characteristics of Paraloid B-72 before aging clearly reflect its chemical composition and confirm its typical properties as an acrylic ester material (Figure 9a; Table 4). The C–H stretching vibration at 2987 cm^−1^ originates from methyl (CH_3_) and methylene (CH_2_) groups, which are typical features of acrylate and methacrylate polymers, indicating that B-72 is a copolymer of methyl acrylate and methyl methacrylate. The stretching vibration of the ester carbonyl group (C=O) at 1735 cm^−1^ further confirms the presence of ester bonds, a core characteristic peak of acrylate polymers [66]. The asymmetric C–O–C stretching vibration at 1155 cm^−1^ and the CH_2_/CH_3_ bending vibration at 1465 cm^−1^ reveal the material’s typical ester and saturated hydrocarbon structures. The peak at 760 cm^−1^ arises from bending vibrations of terminal or branched CH_2_/CH_3_ groups, further supporting the existence of its branched chemical structure [67].

After aging, several measurable spectral variations were observed (Figure 9a; Table 5. The O–H stretching band at 3744 cm^−1^ increased in intensity (from weak to medium), indicating enhanced moisture uptake and/or the formation of hydroxyl-containing species during cyclic aging. Meanwhile, the ester carbonyl band at 1735 cm^−1^ remained strong but became sharper, suggesting that the ester functionality was largely retained, while subtle changes in chain packing and/or local chemical environments may have occurred. The absorption near 1640 cm^−1^ also intensified (from weak to medium). In addition, both the C–O–C stretching band at 1155 cm^−1^ and the CH_2_/CH_3_ bending band at 1465 cm^−1^ showed moderate enhancement, whereas the C–H stretching band at 2987 cm^−1^ remained essentially unchanged. Overall, the FTIR results indicate that Paraloid B-72 exhibits relatively high chemical stability under the applied cyclic aging conditions, with the dominant spectral changes primarily associated with moisture-related effects and limited oxidation-induced structural evolution, rather than pronounced ester cleavage or severe polymer chain scission.

The main component of the Beijing Chemical Works 502 adhesive is ethyl α–cyanoacrylate monomer, the primary constituent of cyanoacrylate adhesives. In the FTIR spectrum (Figure 9b; Table 5), the characteristic strong absorption peak observed at approximately 2240 cm^−1^ is assigned to the stretching vibration of the cyano group (–C≡N), a distinctive marker for α-cyanoacrylate compounds [44,50]. The absorption band at around 1750 cm^−1^ corresponds to the ester carbonyl (C=O) stretching vibration, indicating the presence of acrylate ester structures within the adhesive [68]. In addition, the broad absorption region between 1250 and 1000 cm^−1^ is attributed to ester-related C–O/C–O–C stretching vibrations (C–C–O/O–C–C coupled modes), which is typical for ester–containing polymers.

Compared to Paraloid B-72, the cyanoacrylate adhesive exhibited more evident aging-induced chemical evolution. After aging, a broad O–H stretching envelope intensified in the 3500–3200 cm^−1^ region, indicating increased moisture uptake and/or the formation of hydroxyl-containing species. Meanwhile, the intensities of the C≡N band (~2240 cm^−1^) and the ester carbonyl band (~1750 cm^−1^) remained essentially stable, suggesting that the nitrile and ester functionalities were largely retained. In contrast, the ester-related C–O region (1250–1000 cm^−1^) showed an overall decrease in intensity, implying changes in the ester microenvironment, potentially associated with moisture-induced hydrolysis and hydrogen-bonding interactions during aging [50].

As aging progressed, environmental moisture likely promoted partial hydrolysis of ester groups, generating polar products, which contributes to the enhanced O–H band. The increased hydrogen-bonding interactions among these polar species may induce physical densification of the polymer network. Although such densification can lead to a moderate increase in hardness, it also reduces toughness and interfacial reliability, consistent with the observed embrittlement and debonding behavior in the thin-film region. Overall, these aging-induced chemical and physical changes may adversely affect the long-term stability of cyanoacrylate-based repairs in cultural heritage conservation.

## 4. Discussion

The aging experiments revealed pronounced differences in performance evolution among the investigated adhesives, as well as notable variations arising from different formulations and modification strategies, even within the same adhesive category. These results indicate that adhesive aging behavior is highly sensitive to material composition and structural design. Owing to experimental constraints, direct mechanical testing of bonded joints, such as tensile or shear strength measurements, was not conducted in this study. Nevertheless, the pencil scratch test, as a macroscopic hardness evaluation method, provides an intuitive and comparative assessment of aging-induced physical property changes in adhesive coatings. The observed hardness evolution reflects underlying molecular and structural transformations during aging, offering meaningful insight into material performance trends.

While direct adhesion strength measurements were beyond the scope of the present study, the systematic combination of hardness evolution, interfacial morphology, and FTIR-detected network densification provides converging evidence for relative mechanical performance trends under identical aging conditions. Future work will focus on incorporating quantitative mechanical parameters, including elastic modulus and brittleness, to establish a more comprehensive understanding of long-term adhesive stability.

### 4.1. Epoxy Resins

Colorimetric measurements reveal that both epoxy resin adhesives, Hezhong AAA and Hongxing 509, undergo pronounced and progressive color changes during cyclic aging. As shown in Figure 6a and Appendix A, the total color difference (ΔE) of Hezhong AAA increased continuously from 0 to 17.62 after 15 cycles, while Hongxing 509 reached a lower final ΔE of 11.51. In both cases, the color variation is dominated by a substantial increase in the b* parameter, whereas changes in L* remain relatively limited, indicating that yellowing rather than darkening constitutes the primary aging-related visual degradation. The temporal evolution of ΔE shows a rapid increase during the first several cycles followed by a slower growth phase, suggesting that early-stage photo-oxidative reactions play a dominant role, while later aging stages are controlled by diffusion-limited oxidation processes. The chromaticity distribution in Figure 6b further confirms that the color shift is concentrated in the 570–580 nm wavelength range, which is characteristic of epoxy resin yellowing [53].

Hardness measurements (Figure 7) demonstrate a concurrent increase in coating hardness for both epoxy adhesives. Hezhong AAA exhibits a transition from 4B in the unaged state to approximately 3H after aging, whereas Hongxing 509 increases from an initial 2H to approximately 5H, remaining the hardest material throughout the experiment. This continuous hardening trend indicates progressive densification of the cross-linked network during aging [60].

FTIR spectra (Figure 8; Table 2 and Table 3) provide molecular-level evidence supporting these macroscopic observations. The emergence and intensification of carbonyl absorption bands at ~1730–1745 cm^−1^ indicate oxidative formation of C=O–containing species, which are widely recognized as chromophoric structures responsible for epoxy yellowing. Simultaneously, the weakening of epoxy-related C–O–C absorption peaks suggests continued post-curing and network rearrangement. The relatively stable Si–O–Si absorption in Hongxing 509 correlates with its lower ΔE values and indicates that silicone modification contributes to improved resistance against oxidative degradation [63].

Taken together, the combined colorimetric, hardness, and FTIR data demonstrate that epoxy resin adhesives exhibit strong structural stability during aging but at the cost of significant visual alteration and increased rigidity.

### 4.2. Acrylic Resins

In contrast to epoxy resins, Paraloid B-72 exhibits minimal color change throughout the aging process. As shown in Figure 6a and Appendix A, ΔE values remain consistently below 2, and variations in L*, a*, and b* do not show a systematic trend with aging cycles. Within the accuracy limits of instrumental color measurement, these fluctuations can be regarded as negligible, confirming excellent optical stability under simulated aging conditions.

Despite its visual stability, morphological observations (Figure 4) reveal pronounced early-stage physical changes. Significant variations in bubble size and morphology occur during the initial aging cycles, followed by gradual stabilization. These changes correspond to the solvent-based curing mechanism of B-72, in which residual acetone continues to evaporate after apparent surface solidification. The sequential evolution of individual bubbles (Figure 4e–i) quantitatively illustrates this delayed stabilization process [43].

Hardness measurements (Figure 7) show a moderate increase from 5B to approximately H after aging, indicating limited network stiffening compared to epoxy resins. FTIR analysis (Figure 9a; Table 4) shows that the ester carbonyl band at 1735 cm^−1^ remains strong and becomes sharper after aging, while the C–O–C band at 1155 cm^−1^ slightly intensifies. These changes suggest limited chemical evolution and are consistent with the moderate hardness increase.

Therefore, the data indicate that Paraloid B-72 maintains excellent color stability but exhibits sensitivity to thermal and hygrothermal conditions, which primarily manifests as delayed physical stabilization rather than pronounced chemical discoloration [16].

The cyanoacrylate adhesive 502 demonstrates a markedly different aging pattern. Colorimetric data (Figure 6a; Appendix A) show that ΔE values remain below 2 throughout the experiment, comparable to Paraloid B-72, indicating high resistance to visible discoloration.

However, hardness measurements (Figure 7) reveal a gradual increase from 5B to approximately B, although the magnitude of hardening is smaller than that observed for epoxy resins. Morphological observations (Figure 5c) provide critical insight into performance degradation: despite the absence of obvious surface deterioration in the thick adhesive region, embrittlement and interfacial debonding occur in the thin-film region.

FTIR spectra (Figure 9b; Table 5) show intensified absorption of cyano (C≡N) and carbonyl (C=O) groups, along with the emergence of broad O–H stretching bands [45]. These changes indicate moisture-induced ester hydrolysis and subsequent hydrogen-bond-mediated network densification. While such molecular rearrangement increases hardness, it simultaneously reduces toughness and interfacial adhesion, explaining the observed detachment from the substrate.

Importantly, the degradation of bonding performance precedes any significant visual change, demonstrating that color stability alone is insufficient to assess a long term reliability of cyanoacrylate adhesives in conservation contexts.

### 4.3. Comparing

From the fundamental properties observed prior to aging, epoxy resin adhesives exhibit significantly greater strength compared to acrylic resin-based materials. Specifically, Hezhong AAA and Hongxing 509 demonstrate far higher hardness during the initial curing stage than B-72 and 502. Except for Hongxing 509, the other three adhesives appear transparent in their unaged state, meeting the visual requirements for bonding transparent glaze layers in porcelain restoration. However, distinct differences emerge after aging: epoxy resin-based adhesives show a significant increase in color difference, while acrylate-based adhesives maintain essentially unchanged color stability. Regarding hardness variation, all adhesives exhibit varying degrees of hardening, but adhesive 502 demonstrates interfacial debonding from the glass slide during the hardening process, indicating a significant deterioration in its bonding performance. Fourier transform infrared spectroscopy (FTIR) analysis reveals that the aging of epoxy resins is primarily influenced by ultraviolet radiation, leading to the formation of chromophores with visible-light absorption capacity and resulting in noticeable discoloration. The aging of Paraloid B-72, however, is mainly driven by ambient temperature and humidity fluctuations, with its relatively poor heat resistance causing internal bubbles within the adhesive layer to undergo a sequence of shrinkage, stabilization, cracking, fragmentation, and eventual integration. For adhesive 502, molecular rearrangement and cross-linking occur under humid conditions, leading to increased hardness accompanied by reduced toughness, along with the manifestation of interfacial debonding.

The performance evolution of different adhesive types under aging conditions is collectively influenced by their chemical structures, environmental factors, and inherent material properties, reflecting the diversity and complexity of their aging behaviors.

### 4.4. Application

Based on the performance differences among the adhesives, targeted selection and combined use can be applied in practical restoration. Epoxy resin adhesives, owing to their high hardness, moderate curing speed, and good structural support capacity, are more suitable for load-bearing parts of artifacts or structural bonding requiring long-term stability. And the trade-off with their potential for color change must be considered. Paraloid B-72, valued for its good reversibility, lower mechanical impact, and ease of secondary removal, is well-suited for auxiliary bonding or protective coating layers [14]. Adhesive 502, with its rapid curing and color stability, is appropriate for quick on-site reassembly and temporary fixation of fragments during archaeological excavation or the preliminary stages of restoration due to its operational convenience. However, it is not recommended for long-term use and should be replaced with cultural relic adhesives offering higher stability during the formal restoration phase.

In summary, different adhesive types possess distinct advantages and limitations in terms of mechanical properties, aging stability, reversibility, and visual effect. Through scientific material selection, rational combination, and targeted application, it is possible to ensure both bonding strength and operational flexibility while achieving the long-term safety and aesthetic integrity of cultural relic conservation and restoration. This approach provides an actionable strategy and practical basis for material selection.

## 5. Conclusions

This study systematically investigated the aging behavior of four adhesives commonly used in the restoration of porcelain artifacts in Chinese museums through simulated cyclic aging experiments. By integrating surface morphological observations, colorimetric evolution, hardness variation, and Fourier transform infrared spectroscopy (FTIR) analysis, the performance evolution characteristics and dominant aging mechanisms of different adhesive systems under complex environmental conditions were clarified.

The results indicate that epoxy resin adhesives exhibit high structural stability and significant hardness enhancement during aging but are prone to oxidative discoloration due to the formation of carbonyl-containing chromophores. Silicone-modified epoxy formulations show improved chemical stability, although increased rigidity may introduce potential risks to fragile ceramic substrates. Paraloid B-72 maintains excellent color stability and reversibility throughout aging, confirming its suitability for visually sensitive and reversible conservation treatments despite limited thermal resistance and delayed physical stabilization. In contrast, cyanoacrylate adhesive 502, while visually stable and operationally efficient, undergoes moisture-induced molecular rearrangement leading to embrittlement and interfacial debonding, rendering it unsuitable for long-term structural conservation. These findings support a functionally differentiated adhesive selection strategy tailored to structural demands, visual requirements, and conservation objectives.

It should be noted that this study has certain limitations. Direct adhesive-substrate adhesion strength measurements were not conducted, and mechanical performance was primarily interpreted in terms of relative aging trends rather than absolute values. In addition, the use of glass substrates and accelerated aging conditions cannot fully replicate real ceramic interfaces or long-term natural service environments. Accordingly, the conclusions are intended for comparative assessment of adhesive aging behavior under identical conditions rather than direct prediction of service lifetimes. Future studies incorporating ceramic substrates, direct adhesion strength testing, and combined natural and artificial aging approaches will further enhance the applicability of these findings to conservation practice.

## Figures and Tables

**Table 1:** Selection of Adhesives.

Product Name	Adhesive Type	Manufacturer
Hezhong AAA	Epoxy resin	Zhejiang Huangyan Guanghua Adhesive Factory, Taizhou, China
HongXing 509	Epoxy resin	Cixi Tiandong Adhesive Factory, Ningbo, China
Paraloid B-72	Acrylic resin	Dow Chemical Company, Midland, MI, USA
502	Cyanoacrylate	Beijing Chemical Works, Beijing, China

**Table 2:** FTIR characteristic absorption bands of Hezhong AAA epoxy adhesive before and after aging.

Wavenumber/cm^−1^	Fun. Group	Before Aging	After Aging	References
3500–3200	O–H stretch	broad	broad	[51,52]
3050	C–H stretch	medium	medium	[51,61]
1607, 1510	C=C stretch	medium	medium–strong	[52]
1730	C=O stretch	weak	strong	[53,62]
1240	C–O–C stretch	medium	medium–strong	[61]
830	p-substituted aromatic C–H (out–of–plane)	strong	strong	[61]

**Table 3:** FTIR characteristic absorption bands of Hongxing 509 epoxy adhesive before and after aging.

Wavenumber/cm^−1^	Fun. Group	Before Aging	After Aging	References
3676	Si–OH stretch	sharp	sharp	[63,64]
3500–3200	O–H stretch	broad	broad	[51,52]
2950–2850	(–CH_3_, –CH_2_–)	medium	medium	[53]
1745	C=O stretch	weak	medium	[53,62]
1608,1508	C=C stretch	medium	medium	[52]
1245	C–O–C stretch	medium	medium	[65]
1015	Si–O–Si stretch	strong	strong	[63,64]
825	p–substituted aromatic C–H (out–of–plane)	medium	weak	[61]

**Table 4:** FTIR characteristic absorption bands of Paraloid B-72 before and after aging.

Wavenumber/cm^−1^	Fun. Group	Before Aging	After Aging	References
3744	O–H stretching	weak	medium	[66]
2987	C–H stretching (CH_3_, CH_2_)	medium	medium	[67]
1735	C=O stretching	strong	strong, sharp	[66]
1640	C=C stretch	weak	medium	[67]
1465	CH_2_/CH_3_ bending vibration	medium	medium–strong	[66]
1155	C–O–C asymmetric stretching	medium	medium–strong	[66]

**Table 5:** FTIR characteristic absorption bands of Beijing Chemical Works 502 before and after aging.

Wavenumber/cm^−1^	Fun. Group	Before Aging	After Aging	References
3500–3200	O–H stretch	weak	medium, broad	[50]
2240	C≡N stretch	medium	medium	[50]
1750	C=O stretch	strong	strong	[68]
1250,1150,1009	C–O/C–O–C stretching	medium–strong	medium	[50,68]

## Data Availability

The original contributions presented in this study are included in the article. Further inquiries can be directed to the corresponding authors.

## Appendix A

**Table A1:** Colorimetric Changes of Adhesives Before and After Aging.

Cycles Aging	HeZhong AAA				HongXing 509			
	L*	a*	b*	ΔE	L*	a*	b*	ΔE
0	62.61	−2.57	0.18	0.00	63.99	−2.45	0.58	0.00
1	62.83	−4.22	4.47	4.60	63.15	−3.82	4.43	4.17
2	63.21	−4.63	8.86	8.94	62.42	−3.52	5.62	5.39
3	63.05	−5	10.43	10.54	63.08	−4.26	8.11	7.80
4	61.84	−4.95	11.34	11.44	62.52	−5.13	9.25	9.19
5	61.47	−5.29	12.64	12.80	63.77	−4.53	9.31	8.98
6	61.78	−5.24	12.93	13.05	63.09	−5.01	9.46	9.29
7	61.89	−5.31	13.38	13.50	61.86	−5.04	9.52	9.55
8	61.13	−5.5	14.59	14.78	62.9	−5.41	10.88	10.77
9	62.13	−5.47	15.19	15.30	61.96	−5.02	10.05	10.02
10	61.72	−5.8	15.49	15.67	60.37	−4.99	10.34	10.72
11	60.46	−5.63	16.16	16.41	62.21	−5.01	10.93	10.81
12	60.97	−5.53	16.43	16.60	61.5	−5.43	11.08	11.20
13	61.22	−5.79	17.06	17.24	61.9	−4.67	11.47	11.31
14	61.45	−5.68	17.12	17.26	61.96	−4.94	11.62	11.50
15	61.74	−5.56	17.52	17.62	61.31	−5.22	11.43	11.51
**Cycles** **aging**	**Beijing Chemical Works 502**				**Paraloid B72**			
	**L***	**a***	**b***	**ΔE**	**L***	**a***	**b***	**ΔE**
0	64.52	−2.43	0.39	0.00	65.41	−2.5	0.46	0.00
1	64.22	−2.75	0.86	0.64	64.41	−1.8	0.22	1.24
2	63.98	−2.65	0.96	0.82	64.23	−2.59	0.44	1.18
3	64.03	−2.93	1.32	1.16	64.55	−2.66	0.69	0.90
4	64.6	−2.92	1.47	1.19	64.48	−2.62	0.17	0.98
5	64.1	−2.95	1.6	1.38	64.44	−2.65	0.44	0.98
6	64.16	−2.93	1.96	1.69	64.91	−2.31	1.62	1.28
7	64.51	−2.76	1.27	0.94	65.04	−2.52	0.65	0.42
8	63.22	−2.12	1.65	1.84	64.64	−2.61	0.63	0.80
9	64.64	−2.03	1.84	1.51	65.27	−2.56	0.74	0.32
10	63.89	−2.9	1.27	1.18	64.07	−2.65	0.39	1.35
11	63.86	−3.02	1.68	1.56	63.83	−2.6	0.63	1.59
12	64.4	−3.03	1.98	1.70	64.49	−2.61	0.63	0.94
13	64.4	−2.95	1.88	1.58	64.3	−2.42	0.49	1.11
14	64.75	−3.03	1.92	1.66	64.16	−2.49	0.54	1.25
15	64.8	−3.02	2.14	1.87	64.07	−2.4	0.74	1.37

Cycles aging denotes the number of cyclic aging treatments. HeZhong AAA, HongXing 509, Beijing Chemical Works 502, and Paraloid B-72 indicate the investigated adhesive types. L*, a*, and b* are the CIE L*a*b* color parameters, and ΔE represents the color difference at each aging cycle relative to the initial state (0 cycle).
